# Nano-/Microfabrication of Biomaterials

**DOI:** 10.1155/2014/963972

**Published:** 2014-05-27

**Authors:** Inn-Kyu Kang, Yoshihiro Ito, Oh Hyeong Kwon

**Affiliations:** ^1^Department of Polymer Science and Engineering, Kyungpook National University, Daegu 702-701, Republic of Korea; ^2^Nano Medical Engineering Laboratory, RIKEN, Saitama 351-0198, Japan; ^3^Department of Polymer Science and Engineering, Kumoh National Institute of Technology, Gumi 730-701, Republic of Korea

The surface composition and functionality of biomaterials and scaffolds are the most important factors for their applications in biomedical fields and tissue regeneration. Bulk and surface properties of biomaterials influence vital cell activities such as cell adhesion, proliferation, extracellular matrix secretion, and differentiation. Nano-/microstructural control is found to be another key factor in tailoring the bioactivity of material surfaces. The morphological/topographical designing of the grafts is also proved to be a strategic approach in improving the bioactivity and biological responses of the biomaterial. Furthermore, the nano-/microstructured grafts possess higher specific surface area, which will provide much more adsorption sites to adsorb bioactive molecules.

In view of these specialties of the biomaterials, several investigators are invited to contribute original research findings that can stimulate continuing efforts to understand the dimensions of the polymers and bioceramics as well as their composites essentially with bioactive compositions along with 2D/3D nano-/microlevel surface structures. The special issue is divided into three categories based on the key words of nanoparticles, surface control, and 3D scaffolds. The published works are briefly addressed as follows.

In the category of nanoparticles, S.-J. Han et al. prepared herceptin-immobilized CdSe/ZnS core-shell quantum dots (QDs). Mean size of the quantum dots (28 nm) as determined by dynamic light scattering was increased up to 86 nm after herceptin immobilization. It was found, from* in vitro* cell culture experiment, that keratin forming cancer cells (KB) were well proliferated in the presence of herceptin-conjugated QDs (QD-Her) while most of breast cancer cells (SK-BR3) were dead. The data from confocal laser scanning microscope showed that the QD-Her specifically bound to the membrane of SK-BR3 and almost saturated after 6 hours of incubation. This result suggested that growth signal of the breast cancer cell is completely inhibited by specific binding of the herceptin to the Her-2 receptor of SK-BR3 membrane, resulting in cell death. A. Takahashi et al. reported impact of core-forming segment structure on drug loading in biodegradable polymeric micelles using PEG-*b*-poly(lactide-*co*-depsipeptide) block copolymers. As a result, the drug loading increased with increase in the mole fraction of depsipeptide unit in the hydrophobic segments. Y. Huang et al. prepared reduction-triggered breakable polymeric micelles incorporated with methotrexate (MTX) using amphiphilic PAA-g-PEG copolymers having S–S bonds in the backbone. The drug loading content and drug loading efficiency increased along with more hydrophobic segments in the copolymers. In reductive environments, the entire MTX payload could be quickly released due to the reduction-triggered breakage of the micelles.

In the category of surface control, J. Kang et al. immobilized bone morphogenetic protein (BMP) on DOPA- or dopamine-treated titanium surfaces to enhance osseointegration. The immobilized BMP induced specific signal transduction and alkali phosphatase, a differentiation marker. J. O. Eniwumide et al. investigated the potential of a novel micropatterned substrate for neocartilage formation. In the comparison of flat and honeycomb-patterned surface, accumulation of DNA and keratin sulphate was higher on the honeycomb surface, suggesting potential usefulness of honeycomb-based scaffolds during early cultures of neocartilage and engineered soft tissue. T.-Y. Kwon et al. examined the polymerization shrinkage of five dental modeling resins as well as one temporary PMMA/MMA resin (control). They concluded, from the study of final volumetric shrinkage values for the modeling resins, that the optimal control of the polymerization kinetics seems to be more important for producing high-precision resin structures rather than using dental modeling resins. S. W. Hong et al. studied enhanced neural cell adhesion and neurite outgrowth on graphene-based biomimetic substrates. The result implied that graphene and CNTs, even though they were the same carbon-based nanomaterials, show differential influences on neural cells. Furthermore, graphene-coated or -patterned substrates were shown to substantially enhance the adhesion and neurite outgrowth of PC-12 cells. M. J. Kim et al. synthesized and evaluated biodegradable and elastomeric polyesters (poly(glycerol sebacate) (PGS)) using polycondensation between glycerol and sebacic acid to form a cross-linked network structure without using exogenous catalysts. They reported that synthesized materials possess good mechanical properties and elasticity and surface erosion biodegradation behavior and that the surface morphology and thickness of coating layer could be controlled by adjusting the electrospraying conditions and solution parameters.

In the category of 3D scaffolds, X. He et al. prepared cylinder-shaped porous sponges of poly(L-lactic acid), poly(DL-lactic-co-glycolic acid), and poly(*ε*-carprolactone). SEM observation showed that the cylinder-shaped sponges had evenly distributed bulk pore structures and the wall surfaces were less porous with a smaller pore size than the wall bulk pore structures. The porosity and pore size of the sponges could be controlled by the ratio and size of the porogen materials. They also studied collagen scaffolds with controlled insulin release and controlled pore structure for cartilage tissue engineering. Collagen-microbead hybrid scaffold was prepared by hybridization of insulin loaded PLGA microbeads with collagen using a freeze-drying technique. The pore structure of the hybrid scaffold was controlled by using preprepared ice particulates having a diameter range of 150–250 *μ*m. Hybrid scaffold had a controlled pore structure with pore size equivalent to ice particulates and good interconnection. Culture of bovine articular chondrocytes in the hybrid scaffold demonstrated high bioactivity of the released insulin. The hybrid scaffold facilitated cell seeding and spatial cell distribution and promoted cell proliferation. H. H. Oh et al. fabricated and characterized thermoresponsive polystyrene nanofibrous mats for cultured cell recovery. Cultured cells were easily detached from the PIPAAm-grafted surfaces by reducing culture temperature to 20°C, while negligible cells were detached from ungrafted surfaces. Moreover, cells on PIPAAm-grafted PS nanofibrous mats were detached more rapidly than those on PIPAAm-grafted PS dishes. Y. I. Yoon et al. fabricated nano-/microfibrous scaffolds using melt and hybrid electrospinning and surface modification of poly(L-lactic acid) with plasticizer. The silk fibroin (SF)/PLA (20/80) scaffolds consisted of a randomly oriented structure of PLA microfibers (average fiber diameter = 8.9 *μ*m) and SF nanofibers (average fiber diameter = 820 nm). The PLA nano-/microfiber (20/80) scaffolds were found to have pore parameters similar to the PLA microfiber scaffolds. The PLA scaffolds were treated with plasma in the presence of either oxygen or ammonia gas to modify the surface of the fibers. They claimed that the control of surface property and fiber diameter could be useful in the design and tailoring of novel scaffolds for tissue engineering.

*Inn-Kyu Kang* *Yoshihiro Ito* *Oh Hyeong Kwon*

